# Geographical Variations in Patients with Heart Failure and Preserved Ejection Fraction: A Sub-Group Analysis of the APOLLON Registry

**DOI:** 10.4274/balkanmedj.galenos.2019.2019.2.17

**Published:** 2019-07-11

**Authors:** Bülent Özlek, Eda Özlek, Hicaz Zencirkıran Ağuş, Mehmet Tekinalp, Serkan Kahraman, Oğuzhan Çelik, Cem Çil, Özcan Başaran, Volkan Doğan, Bedri Caner Kaya, İbrahim Rencüzoğulları, Altuğ Ösken, Lütfü Bekar, Mustafa Ozan Çakır, Yunus Çelik, Kadir Uğur Mert, Kadriye Memiç Sancar, Samet Sevinç, Gurbet Özge Mert, Murat Biteker

**Affiliations:** 1 Department of Cardiology, Muğla Sıtkı Koçman University Training and Research Hospital, Muğla, Turkey; 2 Clinic of Cardiology, İstanbul Mehmet Akif Ersoy Thoracic and Cardiovascular Surgery Training and Research Hospital, İstanbul, Turkey; 3 Clinic of Cardiology, Kahramanmaraş Necip Fazıl City Hospital, Kahramanmaraş, Turkey; 4 Clinic of Cardiology, Şanlıurfa Mehmet Akif İnan Training and Research Hospital, Şanlıurfa, Turkey; 5 Department of Cardiology, Kafkas University School of Medicine, Kars, Turkey; 6 Clinic of Cardiology, İstanbul Dr. Siyami Ersek Thoracic and Cardiovascular Surgery Training and Research Hospital, İstanbul, Turkey; 7 Clinic of Cardiology, Hitit University Çorum Erol Olçok Training and Research Hospital, Çorum, Turkey; 8 Department of Cardiology, Zonguldak Bülent Ecevit Universiy School of Medicine, Zonguldak, Turkey; 9 Clinic of Cardiology, Yüksek İhtisas Hospital, Kırıkkale, Turkey; 10 Department of Cardiology, Eskişehir Osmangazi University School of Medicine, Eskişehir, Turkey; 11 Clinic of Cardiology, Eskişehir Yunus Emre State Hospital, Eskişehir, Turkey

**Keywords:** Geography, heart failure, outpatients, Turkey

## Abstract

**Background:**

Clinical characteristics of patients with heart failure may vary geographically. However, limited data are available regarding the geographical differences of patients with heart failure and preserved ejection fraction.

**Aims:**

The present subgroup analysis aims to investigate the geographical differences in clinical characteristics, management, and primary etiology of patients with heart failure and preserved ejection fraction in Turkey.

**Study Design:**

A cross-sectional study.

**Methods:**

A comPrehensive, ObservationaL registry of heart faiLure with mid-range and preserved ejection fractiON (APOLLON) is a multicenter and observational study conducted in seven regions of Turkey (NCT03026114). The present study is a post-hoc analysis of the APOLLON registry. In this substudy, we compared the clinical characteristics of 819 consecutive patients with heart failure and preserved ejection fraction (mean age, 67 years; 57.8% women) admitted to cardiology outpatient units in different geographical regions.

**Results:**

Based on the geographical distribution of the entire Turkish population, the highest number of patients enrolled were from Marmara (271 patients, 33.1%). All demographical characteristics, clinical and laboratory findings, comorbidities, primary etiology, and medications prescribed were significantly different between the regions. Furthermore, inter-regional gender differences were identified. Comparatively, the Aegean and Mediterranean regions had older patients with heart failure and preserved ejection fraction (p<0.001), and the Black Sea, Southeast, and East Anatolia regions had predominantly male patients (51.2, 54.5, and 56.9%, respectively; p=0.002). Notably, the Mediterranean and Southeast Anatolia had more symptomatic patients, and history of hospitalization for heart failure was more prevalent in Southeast Anatolia (33.3%, p<0.001). Prevalence of atrial fibrillation was higher in the Mediterranean and Southeast Anatolia regions (51 and 48.5%, p<0.001), and patients with heart failure and preserved ejection fraction had a higher prevalence of hypertension in the Mediterranean, Southeast Anatolia, and Black Sea regions (p=0.002). Angiotensin-converting enzyme inhibitors were more frequently prescribed in East Anatolia (52.3%, p=0.001), and the prevalence of patients with heart failure and preserved ejection fraction using loop diuretics (48.8%, p=0.003) was higher in the Black Sea region.

**Conclusion:**

This study was the first to show geographical differences in clinical characteristics of patients with heart failure and preserved ejection fraction in Turkey. Determination of the clinical characteristics of the heart failure and preserved ejection fraction population based on the geographical region may enables physicians to adopt a region-specific clinical approach toward heart failure and preserved ejection fraction.

Heart failure (HF) with preserved ejection fraction (HFpEF) is a common global public health issue and comprises approximately half of the patient population with HF (1,2). Recent studies have shown that its prevalence ranges from 1.1% to 5.5%, and it is estimated to further increase with greater diagnostic awareness; increased life expectancy; and an increasing prevalence of hypertension, atrial fibrillation, diabetes, and obesity (3,4). Patients with HFpEF have a poor prognosis with a lower survival rate and higher excess mortality than the general population (5). Moreover, the prognosis of patients with HFpEF has not shown any substantial improvement over the past decades (6). Furthermore, previous studies have revealed that the risk factors for HFpEF include advanced age, hypertension, female sex, and physical inactivity (7).

Based on the geographical region, patients with HF may have significant differences in clinical characteristics, medications, rehospitalization frequency, and mortality (8), as documented in previous HF trials (9). However, limited data are available regarding the importance of geographical differences in patients with HFpEF. Among diverse racial/ethnic groups in different geographical regions, an increase in the number of baseline risk factors is associated with an increased risk of HF (10). Most investigations regarding the association between HF and risk factors have been studied at a personal level, which provides essential information to regulate individual health behaviors and improve clinical treatment modalities (11,12). However, risk factor identification and control at the population level across the country can aid in creating a healthy nation. Regional monitoring and recording of modifiable risk factors, the incidence of chronic diseases, and utilization of healthcare services are required to ensure fair provision of healthcare and decrease health disparities across geographical regions according to several health policies (13).

The HAPPY study showed that the prevalence of HF is higher in Turkey than in western countries (14). Turkey is a large country with different ethnicities, seven geographical regions, and an increasing elderly population. The first Geography Congress in Turkey divided it into seven separate regions based on the fauna, human habitat, climate, agricultural diversity, topography, and transportation (15). Marmara, Aegean, Central Anatolia, and the Mediterranean are the western regions and the most socioeconomically developed regions of Turkey. Other regions comprise less socioeconomically developed areas. These regions significantly differ based on the economic growth, demographic characteristics, education, employment rates, and welfare level. Such pronounced geographical differences in living standards of the population can likely reflect the variations in their health status. Moreover, such differences may result in regional differences in the incidence and management of some chronic diseases (16). However, there have been no studies describing regional differences in patients with HFpEF in Turkey. Therefore, we performed a subgroup analysis of the **A** comPrehensive, **O**bservationa**L** registry of heart fai**L**ure with mid-range and preserved ejection fracti**ON** (APOLLON) trial to examine the geographical differences in clinical characteristics, primary etiology, and management of patients with HFpEF in Turkey.

## MATERIALS AND METHODS

### APOLLON study

The present study is a post-hoc analysis of a larger registry of patients with HFpEF and HF with mid-range ejection fraction (HFmrEF). The methodology and results of the APOLLON registry have been published elsewhere (17,18). In brief, APOLLON is a multicenter, cross-sectional, and observational study conducted in seven regions and 12 cities of Turkey (İstanbul, Ankara, Eskişehir, Kayseri, Kırıkkale, Muğla, Kahramanmaraş, Zonguldak, Çorum, Şanlıurfa, Adıyaman, and Kars; ClinicalTrials.gov identifier NCT03026114). The APOLLON registry included 1.065 consecutive outpatients with HFpEF and HFmrEF who were admitted to the outpatient cardiology units of 13 centers across the country with signs and symptoms of HF. The number of participants enrolled from each region was proportional to the population of the relevant region to represent the required geographical diversity. We determined one or more cities and centers where N-terminal pro-B-type natriuretic peptide (NT-proBNP) was available from each geographical region with a high population density and high potential to represent the population of that region. We included educational, research, university, and state hospitals as the study centers to reflect the real-world data of all patients treated in different healthcare settings. The study was conducted between March 31, 2018, and May 20, 2018. All information, such as demographic characteristics; medical history including baseline cardiovascular disease, risk factors, and previous history; and laboratory data including NT-proBNP, electrocardiography, and echocardiography data, were recorded during enrollment. Hypertension, diabetes mellitus, and anemia were defined based on the current guidelines. The estimated glomerular filtration rate was calculated based on the Modification of Diet in Renal Disease equation-4 (19). Chronic kidney disease was defined as estimated glomerular filtration rate <60 mL/min/1.73 m^2^. Ischemic heart disease was detected systematically using a combination of self-report, electrocardiography, review of all available prior medical documents, and clinician contact. Other comorbid conditions were determined based on a review of all available medical records and clinician contact.

Patients were defined as HFmrEF or HFpEF according to the “European Society of Cardiology HF guidelines, 2016” (20). The inclusion criteria for the study were as follows: patients aged ≥18 years, echocardiographic evidence of a left ventricle ejection fraction of ≥40% on admission, at least one additional echocardiographic criterion including evidence of left ventricular diastolic dysfunction or relevant structural heart disease, and patients with signs and symptoms of HF and a NT-proBNP level of >125 pg/mL. All participants were screened using transthoracic echocardiography, and left ventricle ejection fraction was assessed using the modified Simpson’s method. Based on the left ventricle ejection fraction values, the patients were classified into two groups: patients with HFmrEF (left ventricle ejection fraction 40%-49%) and patients with HFpEF (left ventricle ejection fraction ≥50%). At least one additional echocardiographic criterion including the evidence of left ventricular diastolic dysfunction or relevant structural heart disease was required to define HFpEF. According to the guidelines, major structural heart diseases were defined as a left ventricular mass index of ≥95 g/m^2^ for females and ≥115 g/m^2^ for males or left atrial volume index >34 mL/m^2^. Evidence of left ventricular diastolic dysfunction was defined as an E/e′ of ≥13 and a mean e' septal and lateral wall of <9 cm/s.

The exclusion criteria were as follows: a history of corrected valvular heart diseases; primary severe valvular heart disease requiring intervention or surgery; pacemaker implantation or percutaneous coronary intervention in the preceding 30 days; known cardiomyopathy or pericardial constriction; coronary artery bypass graft surgery, stroke (ischemic or hemorrhagic), or myocardial infarction in the preceding 90 days; primary pulmonary hypertension; significant chronic pulmonary disease (e.g., chronic obstructive or restrictive pulmonary disease with severe pulmonary hypertension); heart transplant recipients; cor pulmonale; congenital heart diseases; and pregnancy.

The participating physicians were asked to determine the primary underlying causes of HF development based on the clinical, physical examination, and laboratory findings. The primary etiology of HFpEF was defined based on the following algorithm: “valvular,” if the patient had mild or moderate valvulopathy but no other substantial or uncontrolled risk factor for HF; “hypertensive,” if the patient had resistant or uncontrolled hypertension but no other substantial or uncontrolled risk factor for HF; “ischemic,” if the participant had obstructive coronary artery disease but no other substantial or uncontrolled risk factor for HF; “atrial fibrillation,” if the patient had atrial fibrillation but no other substantial or uncontrolled risk factor for HF. Participants whose primary etiology could not be explained with only one principal cause or could not be clinically determined were categorized as the “other” group.

The APOLLON study was approved by the local institutional review boards of Muğla Sıtkı Koçman University (01.03.2018-01/VI) and informed consent was obtained from all patients.

### Study design and setting

Here, we performed a subgroup analysis of data from the APOLLON study. Using the registry data, we compared the demographic, clinical characteristics, and management of patients with HFpEF in different geographical regions.

### Statistical analysis

The APOLLON registry sample size was calculated based on the assumption that 50% of patients with HF have HFpEF or HFmrEF. Power calculation was based on a two-sided test with a power of 0.80 and significance level α of 0.05; the required sample size was determined to be 1.065 for the entire cohort. One-sample Kolmogorov–Smirnov test was used to determine whether the variables were distributed normally. Based on the data distribution, continuous baseline variables were presented as mean ± standard deviations or median and interquartile range. Non-normally distributed variables were analyzed using the Kruskal–Wallis test, and analysis of variance was used to analyze the normally distributed variables. The categorical variables were expressed in frequencies and percentages (95% confidence interval). Chi-square test was used to analyze the categorical variables. For all analyses, p<0.05 was considered to be statistically significant. Statistical analyses were performed using the statistical package SPSS 24.0 (SPSS Inc, Chicago, Illinois).

## RESULTS

### Study population

The study included 819 patients with HFpEF. The median age of the study population was 67 years, and 57.8% of the patients were females. The geographical distribution of patients with HFpEF was as follows: 271 (33.1%) were from Marmara; 120 (14.7%) were from Aegean; 111 (13.6%) were from Central Anatolia; 102 (12.5%) were from the Mediterranean; 84 (10.2%) were from the Black Sea; 66 (8%) were from Southeast Anatolia; and 65 (7.9%) were from East Anatolia.

### Baseline characteristics and comorbidities based on geographical regions

Baseline characteristics of patients with HFpEF based on geographical regions are listed in Table 1. Regional comparison revealed that the Aegean and Mediterranean regions had older patients with HFpEF; the Black Sea and Southeast and East Anatolia regions had predominantly male patients. In all geographical regions, patients with New York Heart Association class I and II were predominant; however, patients with New York Heart Association class III and IV symptoms, orthopnea, and paroxysmal nocturnal dyspnea were more frequently observed in the Mediterranean and Southeast Anatolia regions than in other regions. Furthermore, pulmonary crepitations, peripheral edema, and history of hospitalization for HF were more common in the Southeast Anatolia region. Patients with HFpEF in Southeast Anatolia had a higher body mass index and systolic and diastolic blood pressure; however, patients in East Anatolia had a lower body mass index and incidence of palpitation and reduced exercise tolerance.

Significant geographical differences were observed in the distribution of comorbid diseases (Table 2). Prevalence of atrial fibrillation was higher in the Mediterranean and Southeast Anatolia regions. Patients with HFpEF had a higher prevalence of hypertension in the Mediterranean, Southeast Anatolia, and Black Sea regions; furthermore, they had a higher prevalence of obstructive sleep apnea in the Aegean and Southeast and East Anatolia regions. In addition, cerebrovascular accident or transient ischaemic attack was more prevalent in Southeast Anatolia. However, patients in the Black Sea region had hyperlipidemia and coronary artery disease more frequently. In contrast, anemia had low prevalence in the Mediterranean region. No significant geographical differences were observed with regard to the prevalence of diabetes mellitus, chronic kidney disease, peripheral artery disease, and chronic obstructive pulmonary disease.

### Regional differences in laboratory parameters and transthoracic echocardiographic findings

Some geographical differences were observed in the distribution of laboratory parameters (Table 3). The NT-proBNP and C-reactive protein levels were significantly higher in Southeast Anatolia. In the Black Sea region, the blood urea nitrogen and uric acid levels were higher and serum ferritin levels were lower than those in other regions.

Region-wise echocardiographic findings are shown in Table 4. Left ventricle ejection fraction was lower in the Aegean region, whereas diastolic functions were significantly worse in the Black Sea region. Patients with HFpEF in Southeast Anatolia had a higher left ventricular end-diastolic and end-systolic dimensions, left ventricular mass index, and prevalence of abnormal left ventricular geometry (concentric hypertrophy). Left atrial volume index was the highest in the Aegean region; however, the incidence of left atrial enlargement was higher in the Aegean and Marmara regions than in other regions. The incidence of mitral regurgitation, aortic stenosis, and aortic regurgitation was significantly lower in East Anatolia, whereas the incidence of aortic stenosis and tricuspid regurgitation was higher in the Aegean and Black Sea regions, respectively.

### Regional differences in management

**Figure 1:**
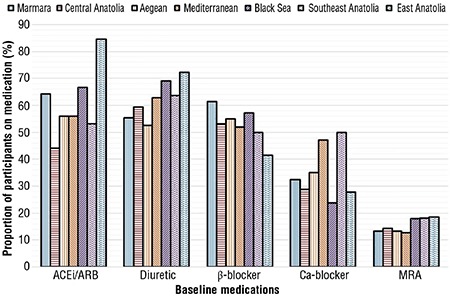
Distribution of medications used for the treatment of heart failure with preserved ejection fraction based on geographical regions. ACEi: angiotensin-converting enzyme inhibitors; ARB: angiotensin receptor blockers; MRA: mineralocorticoid receptor antagonists

Table 5 shows the medication used in each geographical region. Angiotensin-converting enzyme inhibitors (ACEi) were more frequently prescribed in East Anatolia, and nondihydropyridine calcium blockers were used more in Southeast Anatolia. In the Black Sea region, patients with HFpEF were more frequently administered loop diuretics and antiaggregant drugs. However, the use of anticoagulant drugs was high in the Aegean region. No significant geographical differences were observed with regard to other prescriptions (Figure 1).

### Regional differences in the primary etiology of HFpEF

**Figure 2:**
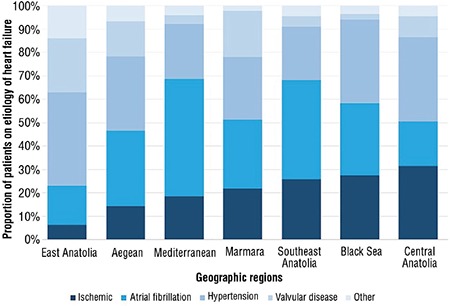
Prevalence of the primary etiology based on geographical regions.

**Figure 3:**
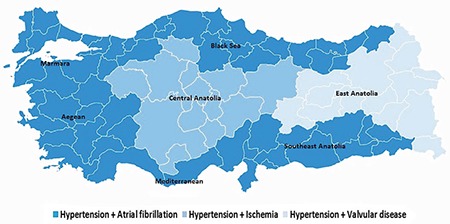
Graphical display of the most common etiology of HFpEF in different geographical regions.

The primary etiology of HFpEF differed between the geographical regions (Figure 2). Atrial fibrillation and hypertension were common causes of HF in the Marmara, Aegean, Mediterranean, Black Sea, and Southeast Anatolia regions. However, hypertension and ischemia were the primary etiologies of HF in Central Anatolia. Notably, hypertension and valvular disease were the primary etiologies of HF in East Anatolia (Figure 3).

## DISCUSSION

The present substudy is a separate analysis of the large and multicenter cohort study investigating the demographic characteristics and clinical findings of patients with HFpEF and HFmrEF in Turkey. We observed substantial variations in the clinical characteristics, medications, and primary etiology in patients with HFpEF enrolled from different geographical regions. Patients with HFpEF were older in the Aegean and Mediterranean regions, were more symptomatic in the Mediterranean and Southeast Anatolia regions, and were more overweight and hypertensive in Southeast Anatolia. The results of our study revealed that hypertension was the most common comorbidity in all geographical regions. The patients living in the Mediterranean region had the highest burden of atrial fibrillation, whereas coronary artery disease was more common in the Black Sea region.

Regional health disparities regarding self-rated health, disease prevalence, and comorbidities have been reported previously. These disparities manifest as geographical differences in cardiovascular health, mortality, and morbidity (21,22). Moreover, the geographical differences in healthcare use across and within countries are well-known (23). In the International Congestive Heart Failure study, Dokainish et al. (24) enrolled 5.823 consecutive patients with HF (66% were clinic outpatients; half of the patients had left ventricle ejection fraction ≥40%) from 108 centers in six geographical regions. They recorded baseline clinical characteristics and followed up patients at 6 months and 1 year from enrollment to record symptoms, clinical findings, drugs, and outcomes. The International Congestive Heart Failure study revealed significant geographical variations in comorbidities (including diabetes mellitus, chronic kidney disease, hypertension, ischemic heart disease, and chronic obstructive pulmonary disease), functional capacity, the prevalence of HF types, principal causes of HF, and medications prescribed. Furthermore, this study showed that New York Heart Association functional class III or IV, previous admission for HF, higher body mass index, the presence of valve disease, chronic kidney disease, and chronic obstructive pulmonary disease were independent predictors of mortality (24). Accordingly, our cohort exhibited significant regional differences in the New York Heart Association functional class, physical examination findings (pulmonary crepitations, peripheral edema, and heart rate), body mass index, prevalence of valvular heart disease, and primary etiology of HFpEF. Furthermore, the incidence of coronary artery disease, atrial fibrillation, hypertension, obstructive sleep apnea, and anemia differed regionally in the APOLLON study. In contrast to the International Congestive Heart Failure study, the prevalence of diabetes mellitus, chronic kidney disease, and chronic obstructive pulmonary disease was similar among regions in our analysis. This difference is probably because the International Congestive Heart Failure study was conducted over a larger geographical area and included all types of HF. A significant difference between our study and some previous HFpEF studies is that our study observed a lower incidence of chronic kidney disease in patients with HFpEF (25,26). This difference may be because the APOLLON registry comprised a younger patient population, and our study excluded hospitalized patients. Furthermore, the APOLLON registry and International Congestive Heart Failure study showed differences in the prescription frequency of HF drugs (24). The use of angiotensin receptor blockers or angiotensin-converting enzyme inhibitors, β-blockers, aldosterone antagonists, loop diuretics, and digoxin were higher in all geographical regions in the International Congestive Heart Failure study.

The “Irbesartan in Heart Failure with Preserved systolic function (I-Preserve)” study included 4.128 patients with HFpEF from different geographical regions (27). In the I-Preserve study, patients from different geographic regions had different clinical characteristics. The proportion of women varied according to the region (from 51.4% in USA/Canada to 70.2% in Latin America); however, the proportion of women was higher in Eastern Europe/Russia than in Western Europe. The I-Preserve study showed that patients in Eastern Europe/Russia had a lower prevalence of atrial fibrillation than in Western Europe or North America. Notably, the prevalence of diabetes mellitus and history of coronary revascularization were the highest in North America. The I-Preserve study reported higher NT pro-BNP and lower estimated glomerular filtration rate levels in the United States/Canada (28). Our study noted a similar difference in gender distribution among the regions. The proportion of women was higher in the Mediterranean and Central Anatolia regions. However, patients in Central and East Anatolia had a lower prevalence of atrial fibrillation than those in the Mediterranean or Southeast Anatolia regions. In the APOLLON registry, NT pro-BNP levels varied based on the region. The highest NT pro-BNP levels were noted in Southeast Anatolia, whereas the lowest levels were in the Black Sea and Marmara regions. Previous studies have shown geographical differences in the crude HFpEF hospitalization rates (28). Concordant with previous data, the APOLLON registry indicated substantial geographical differences in HF hospitalization rates over the past year. The number of patients with HFpEF was the highest in Southeast Anatolia, whereas it was the lowest in the Marmara region. With regard to HF medications, the prescription frequency of loop diuretics, aldosterone antagonists, and digoxin was lower and that of angiotensin-converting enzyme inhibitors or angiotensin receptor blockers was higher in our study than in the I-Preserve study (28). However, both studies showed a similarity regarding the use of β-blockers.

“Candesartan in Heart failure Assessment of Reduction in Mortality and morbidity-preserved” is another significant study that included patients with HFpEF from different geographical areas. The Candesartan in Heart failure Assessment of Reduction in Mortality and morbidity-preserved study compared candesartan with placebo in 3.023 patients with HFpEF (29), and significant geographical differences were observed in the clinical characteristics of patients. Patients were older and had higher systolic blood pressure in Western Europe. In the study population, although patients with New York Heart Association class II were predominant, patients with New York Heart Association class III and IV symptoms were more frequently observed in the United States and Canada. Furthermore, the etiology of HFpEF showed regional variation in the Candesartan in Heart failure Assessment of Reduction in Mortality and morbidity-preserved study, and ischemic etiology was higher in Eastern Europe and Russia (28). In our study, patients with HFpEF in the Aegean and Mediterranean regions were older than the patients in other regions of the country. Patients in the Mediterranean and Southeast Anatolia regions had a higher blood pressure and prevalence of New York Heart Association class III-IV symptoms than patients in other regions. Our study revealed significant regional differences in the patients’ functional capacity, represented by the New York Heart Association functional class. However, the inter-regional differences in the prevalence of valvular diseases, including mitral regurgitation, aortic stenosis, and aortic regurgitation may influence the patients’ volume status and Doppler parameters that are used to predict filling pressures. In addition, the APOLLON registry reported significant inter-regional differences in the primary etiology of HFpEF. In the Candesartan in Heart failure Assessment of Reduction in Mortality and morbidity-preserved study (28), the use of HF medications (including β-blockers, loop diuretics, aldosterone antagonists, angiotensin-converting enzyme inhibitors/angiotensin receptor blockers, and digoxin) varied between different geographic regions. The prescription rates of loop diuretics and digoxin were higher and the use of angiotensin-converting enzyme inhibitors or angiotensin receptor blockers was lower in the Candesartan in Heart failure Assessment of Reduction in Mortality and morbidity-preserved study than in our study. However, both studies showed a similarity in the use of β-blockers and aldosterone antagonists.

This study is a subgroup analysis of APOLLON registry. The primary limitation of the present study is the lack of follow-up data because of the observational and cross-sectional nature of our study. Nevertheless, the design of the study included a large population, all participating physicians collected and recorded data independently, and independent researchers analyzed the data. Therefore, the whole cohort adequately represented real-world patients with HFpEF. However, the findings may not always be applicable in each region because the clinical symptoms and findings were assessed cross-sectionally and the patients were not followed-up prospectively. Although the study sample size was adequate for statistical analysis, a larger sample size may have been more appropriate considering the scope of the study. However, we could not demonstrate causality although we assessed the geographical differences in patients with HFpEF. Another limitation was that the study was limited to outpatient cardiology clinics and did not include hospitalized patients. Moreover, “physician-judged HF” diagnosis concerning the “signs and symptoms of HF” was one of the limitations of the APOLLON registry.

This study was the first multicenter HFpEF registry including patients from seven regions of Turkey that observed marked baseline geographical differences in age, gender, symptoms, clinical characteristics, laboratory findings, background medications, comorbidities, and primary etiology in patients with HFpEF. Despite previous large-scale studies showing the intercontinental differences in the HFpEF population, the APOLLON study revealed that the clinical characteristics of patients may differ regionally even in a relatively small geographical area. However, identifying inter-regional clinical characteristics in the HFpEF population may facilitate a region-specific clinical approach toward HFpEF to determine health service delivery and accelerate the treatment process.

## Figures and Tables

**Table 1:**
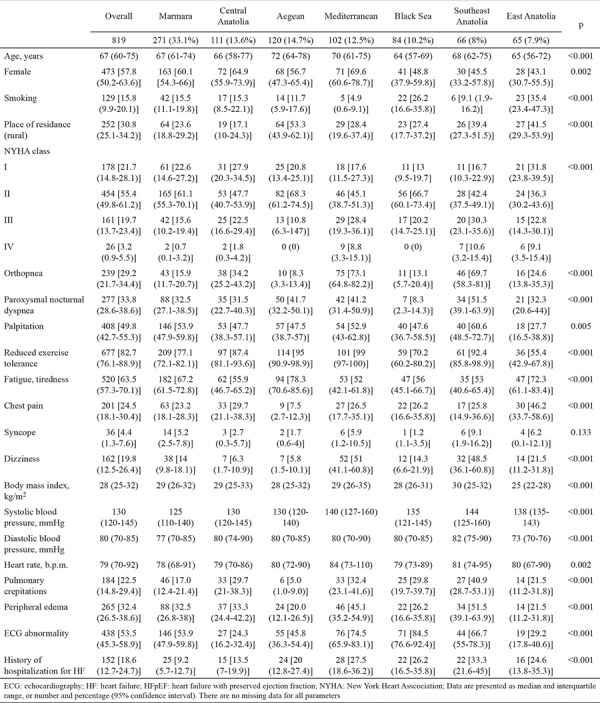
Baseline characteristics of HFpEF patients by geographical regions

**Table 2:**
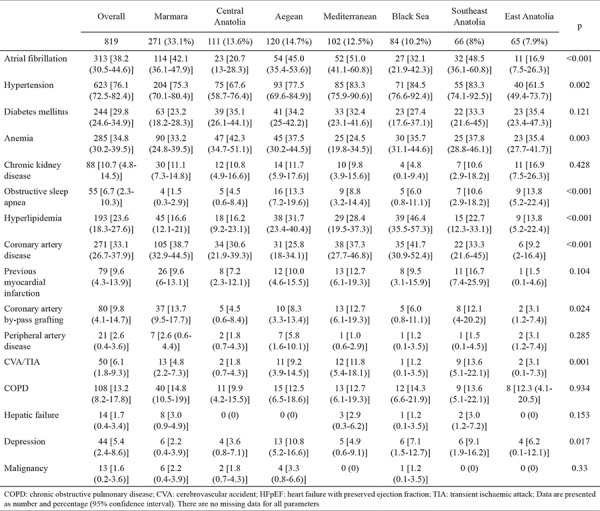
Comorbid conditions in patients with HFpEF by geographical regions

**Table 3:**
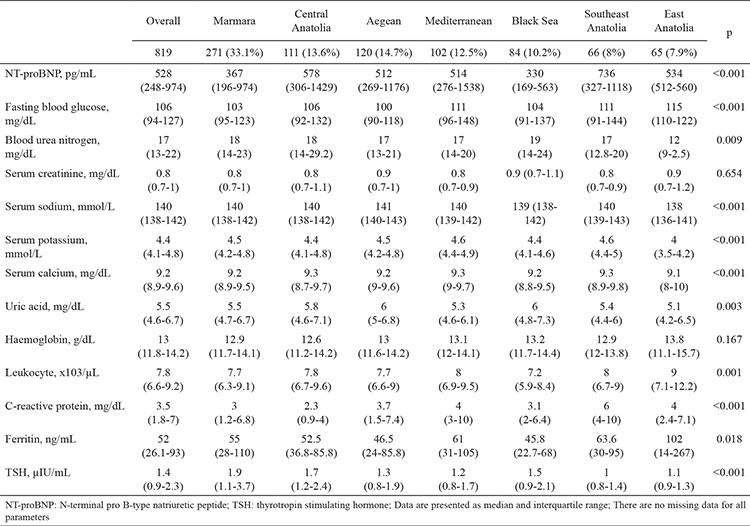
Laboratory parameters

**Table 4:**
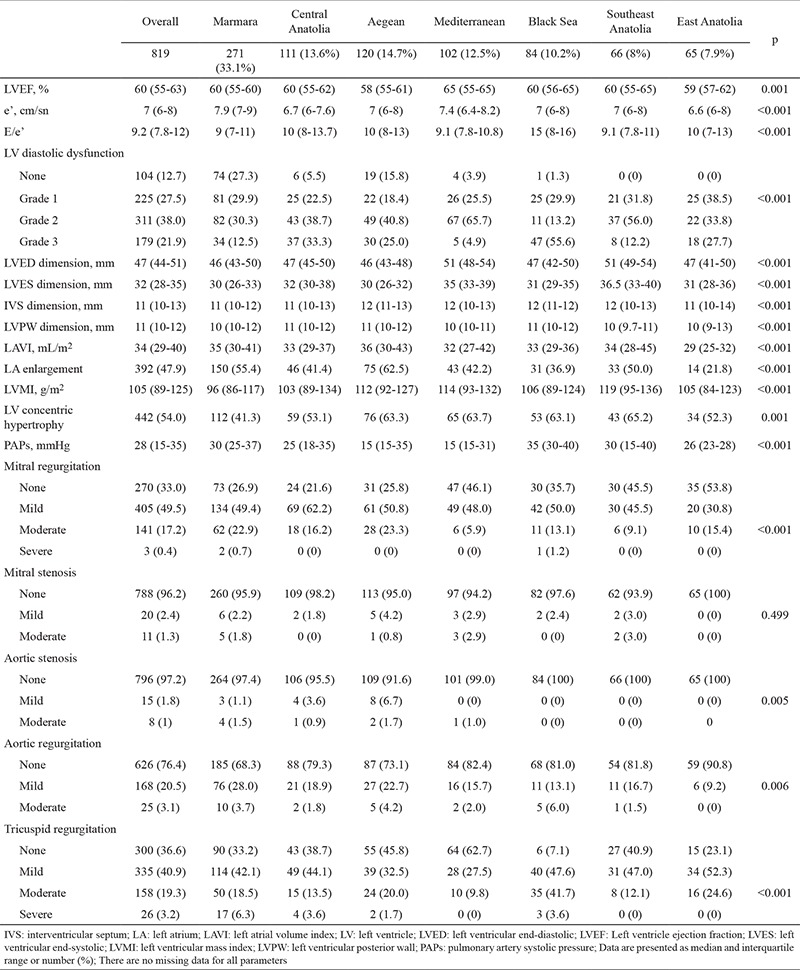
Comparison of the transthoracic echocardiographic findings among the seven geographical regions of Turkey in patients with HFpEF

**Table 5:**
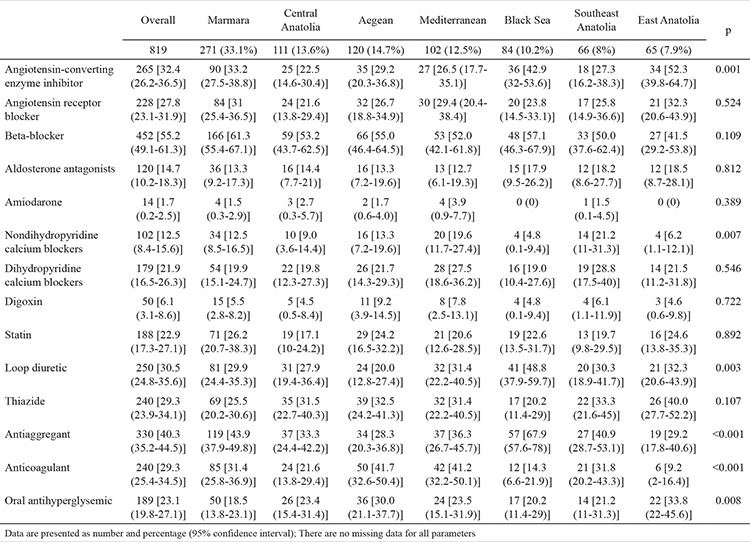
Medications
